# Effects of Child Development Accounts on Parent–Child Educational Engagement and Children’s Hope

**DOI:** 10.3390/children12091136

**Published:** 2025-08-27

**Authors:** Aytakin Huseynli, Jin Huang, Michael Sherraden

**Affiliations:** Center for Social Development, Brown School, Washington University in St. Louis, St. Louis, MO 63130, USA; jhuang23@wustl.edu (J.H.); sherrad@wustl.edu (M.S.)

**Keywords:** asset-building policy, child development accounts, parent–child interaction, educational engagement, children’s hope

## Abstract

**Highlights:**

**What are the main findings?**

- Child Development Accounts (CDAs) have a positive impact on parent–child educational engagement.
- Child Development Accounts (CDAs) have a positive impact on children’s sense of hope.
- These positive effects are most pronounced in the data collected before the COVID-19 pandemic.

**What is the implication of the main finding?**

- Asset-building policies can be an effective strategy for promoting educational engagement and fostering a hopeful, future-oriented mindset among young people.
- Asset-building policies offer a structural approach to reducing psychosocial disparities and to supporting both children and parents simultaneously.

**Abstract:**

**Background:** Child Development Accounts (CDAs) were introduced in the 1990s as a long-term asset-building policy aimed at supporting families in accumulating assets to achieve life goals for their children, including higher education, homeownership, and long-term economic security. Beyond their financial benefits, CDAs have been theorized to strengthen family relationships and improve children’s well-being by fostering a future-oriented mindset and increasing parental involvement in educational activities. **Objective:** This study investigates the impact of CDAs on parent–child educational engagement and children’s sense of hope for the future, contributing to the growing body of research on the multidimensional benefits of asset-based policies for children’s development. **Methods:** Data were drawn from the third wave of the SEED for Oklahoma Kids (SEED OK) study, a rigorous, longitudinal, randomized policy experiment in the United States. The analytic sample comprised 1425 families. Dependent variables were parent–child educational engagement and children’s hope. The independent variable was participation in the SEED OK CDA policy experiment. Baseline sociodemographic variables related to children, mothers, and households were controlled for in the analysis. Multivariate linear regressions and path analysis techniques were employed to assess direct and indirect effects. **Results:** Participation in CDAs was found to improve parent–child educational interactions and enhance children’s hope significantly in the pre-COVID-19 sample. The study’s rigorous design and consistent implementation allowed for establishing causal relationships and long-term developmental benefits. **Conclusions:** CDAs offer not only financial advantages but also contribute meaningfully to strengthening family dynamics and promoting positive psychosocial outcomes for children, supporting their inclusion in comprehensive social policy frameworks.

## 1. Introduction

Fostering parental educational engagement and children’s hope is essential to promoting children’s long-term well-being and academic success. Recent international reports highlight a growing concern over youth well-being and educational disparities in the post-pandemic era [1,2], underscoring the urgency of identifying effective support systems for families. This aligns with a growing international focus on understanding and enhancing child well-being as a multidimensional construct, including psychological, social, and economic resources [3]. While existing research links household assets and wealth to these crucial psychosocial factors, less is known about how asset-building policy interventions can actively shape parental educational engagement and children’s hope. This study leverages longitudinal data from a large-scale, statewide asset-building experiment to examine the impacts of Child Development Accounts (CDAs) on parental engagement and children’s hope. We seek to clarify the role of asset building as a key lever for enhancing the developmental resources that support children’s life outcomes.

### 1.1. The Critical Role of Parental Educational Engagement

Parental educational engagement and interaction with children are increasingly recognized as a critical factor influencing children’s academic trajectories, particularly in the transition to, and completion of, postsecondary education. Postsecondary educational engagement covers multiple dimensions, including academic communication, school-based involvement, and home-based engagement [4,5,6]. Academic communication involves parents’ expectations, values, and the importance they attach to education [7]. School-based involvement includes participation in school activities, parent–teacher conferences, and decision-making processes, while home-based involvement involves assisting with homework [8], maintaining consistent communication about academic matters, and providing necessary resources and emotional support [6].

Studies show that parental engagement during adolescence significantly boosts college aspirations and expectations [8,9]. Wang and Hill [9] found that adolescents who reported frequent parent–child conversations about school and college were more likely to plan to pursue a 4-year degree. These effects were especially pronounced in families with no prior college-going history, suggesting that verbal encouragement and discussions about postsecondary paths can help mitigate the barriers of structural disadvantages. Parents who monitor academic progress, attend school events, and communicate with teachers positively influence their children’s postsecondary readiness. In a longitudinal study by Martínez and Ceballos [10], high school students whose parents engaged regularly in academic monitoring were more likely to complete college-prep coursework and perform better on college entrance exams. This type of involvement provides both accountability and motivation, reinforcing the importance of education and increasing the likelihood of postsecondary enrollment.

Research consistently finds that strong parental educational engagement contributes to reduced rates of school dropout, incarceration, and substance use, while increasing the likelihood of children attaining college degrees and, ultimately, improving their health, employment prospects, and financial security in adulthood [11,12]. The predictive power of parental educational engagement on academic performance is substantial, with large effect sizes reported in multiple studies [13,14,15]. Parental engagement tends to vary by socioeconomic status (SES), with low-SES parents often facing structural barriers to active school involvement. However, interventions aimed at supporting these parents have been shown to enhance their capacity to support their children’s postsecondary goals.

### 1.2. Hope as a Key Psychological Asset

Beyond educational outcomes, empirical research has established that parental educational engagement, alongside socioeconomic factors, such as income, wealth, and education, significantly shapes children’s psychological strengths and socioemotional development, including their hopes, expectations, and behaviors [16,17,18].

Hope, especially among children and youth, is found to be a psychological strength in adverse social conditions [19]. In children, hope means a set of cognitions that set workable goals and come up with pathways to achieve those goals, including essential beliefs about the capacity to move toward those goals [20]. Children with high levels of hope are more effective in achieving the goals they set compared to those with low or no hope. Hope is especially crucial for children and youth navigating adverse social conditions, as it functions as a psychological asset that fosters resilience, life satisfaction, and enhanced well-being [19]. Recent studies confirm that hope is a significant predictor of [21] mental health and academic persistence among youth, functioning as a protective factor against contemporary stressors [21]. Research indicates a strong association between hope and well-being, life satisfaction, and life quality of children.

The focus on hope and engagement fits within a broader conceptual and empirical landscape dedicated to understanding child well-being. International research increasingly emphasizes that child well-being is a multidimensional concept that requires measurement across various domains, including psychological strengths, social connections, and material resources [3,22]. Studies have successfully developed population-level assessments to capture these complex factors, from asset-based approaches in middle childhood to measures of attitudes and behaviors concerning sustainable development [3]. This body of work establishes a clear precedent for examining specific psychosocial factors, like hope, as key indicators of overall well-being.

### 1.3. Connecting Assets, Engagement, and Hope

An important dimension of this discourse involves the role of household asset building in fostering both hope and constructive parent–child interaction. According to the asset-effects theory, asset building is associated with enhanced future orientation and optimism, not only for parents but also for children, as it provides tangible opportunities for education and personal development [23]. Assets and wealth—beyond income—are influential drivers of parental educational engagement and hope. Whether through savings or structured asset-building programs, assets for children nurture parental expectations, reduce stress, and promote behaviors that foster children’s academic success.

Asset-building policies promoting asset accumulation for children play a significant role in strengthening parental engagement in children’s education and in raising parental expectations for children’s academic attainment [23]. These policies have been shown to increase the likelihood that children will enroll in and complete college [24], improve school enrollment rates and academic performance, have positive effects on long-term educational achievement [25], are more likely to pursue higher education, and tend to score higher on standardized tests and exhibit fewer behavioral problems [26]. Especially among low-income families, asset-building policies offer a promising avenue to boost educational outcomes via family engagement.

### 1.4. The Present Study: Objective and Research Question

While the distinct bodies of the literature on parental engagement, children’s hope, and asset-building policies are robust, the linkages between them remain empirically underdeveloped. Specifically, there is a lack of rigorous, experimental evidence demonstrating whether a universal asset-building policy can directly foster these crucial psychosocial resources. The primary objective of this study is, therefore, to determine the effect of a universal asset-building policy on parental educational engagement and children’s hope. Specifically, our research seeks to answer the following question: To what extent do CDAs influence parental engagement and children’s hope within a diverse statewide population?

To address this question, the remainder of this paper is structured as follows. We first provide background on CDAs, the SEED OK policy experiment, and its theoretical underpinnings. We then detail our research methods, including the longitudinal data, sample characteristics, and measures for parental engagement and children’s hope. Subsequently, we present the results from our multivariate regression and path analyses. The paper concludes with a discussion of the findings’ implications for asset-building policy and theory, an acknowledgment of study limitations, and suggestions for future research.

## 2. Background

### 2.1. CDAs as an Asset-Building Policy

As an asset-building policy for children, CDAs were proposed in the 1990s to help families accumulate assets toward life goals for their children, such as postsecondary education, homeownership, business development, and, eventually, retirement security [27]. CDAs are viewed as the centerpiece of a universal, progressive, and potentially lifelong policy model that offers families a structure within which to accumulate assets over time [28]. In the United States, assets in CDAs are often designated for postsecondary education, which can increase the likelihood of college enrollment and completion, especially by groups that have historically not been equally represented in the higher education system [26,29]. CDAs create pathways to college through both financial readiness and identity-building. Qualitative interviews from Maine’s Harold Alfond College Challenge further highlight how CDAs help parents and children envision college as achievable and worthwhile. Thus, it is important to assess the relationships between asset-building policies and parental educational engagement, and children’s hope.

Numerous policies implicitly recognize how household economic resources, including household wealth and assets, shape child development and parent–child interaction. Policy interest in asset-building for children is growing [30]. In the United States, seven states have established statewide policies to encourage asset-building for children [30]. The recently passed H.R.1 Act marks a significant federal development, establishing for the first time a CDA-like policy for all U.S. children under 18 and providing a USD 1000 public initial deposit for those born between 2025 and 2028. Asset-building programs and policies for children have also been implemented in Canada, Israel, Korea, Singapore, Uganda, mainland China, Taiwan, the United Kingdom, and Kazakhstan [12,28].

The design of effective CDAs is guided by a comprehensive framework of ten core principles [28]. Among the most critical are universality, progressivity, and a lifelong perspective [28]. Universality ensures all are included from birth, creating a sense of shared stake and common purpose. Progressivity provides larger initial deposits, or more generous savings matches for disadvantaged children, directly addressing inequality. The lifelong perspective frames the CDA not as an endpoint, but as the foundational account in a system that can integrate with asset-building opportunities throughout an individual’s life, such as for homeownership or retirement [28]. These design elements distinguish them from many other asset-based policies.

### 2.2. Design of the SEED OK Experiment: Population, Setting, and Intervention

**Figure 1:**
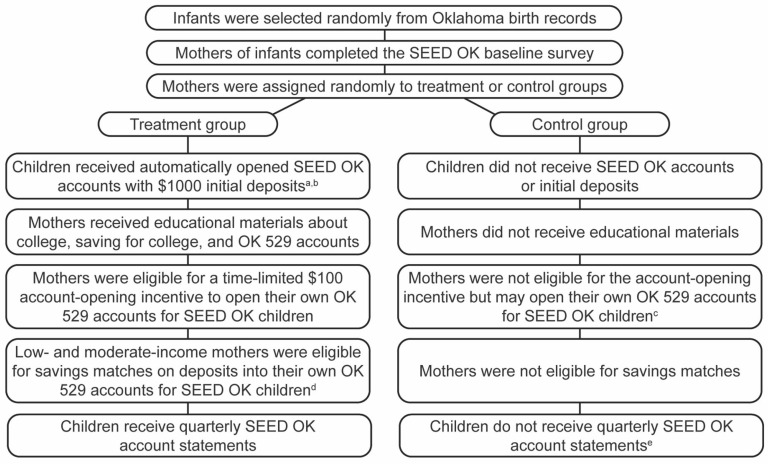
The randomization and intervention components of SEED OK. SEED OK = SEED for Oklahoma Kids; OK 529 = Oklahoma 529 College Savings Plan. Reprinted with permission from Beverly, S. G., Clancy, M., & Sherraden, M. Testing universal college savings accounts at birth: Early research from SEED for Oklahoma Kids (CSD Research Summary No. 14-08). St. Louis, MO: Washington University, 2014, Center for Social Development [33], p. 2. ^a^ One mother opted out of the account for her child for religious reasons. ^b^ Funds are restricted for postsecondary use, to be sent directly to educational institutions. ^c^ Anyone 18 years or older, regardless of their state residency, can open an OK 529 account. ^d^ Savings matches are held in state-owned accounts. ^e^ Because they did not receive automatically opened state-owned accounts, control children do not receive OK 529 account statements, but mothers and other owners of individual OK 529 accounts receive such statements.

SEED for Oklahoma Kids (SEED OK) is the most important policy test for CDAs in the USA, with multimethod, longitudinal, and rigorous experimental, randomized-controlled-trial research [31]. This ongoing large-scale policy test of CDAs for universality, progressivity, and automatic opening started in 2007 in Oklahoma. The sample for SEED OK was selected from the birth records provided by the Oklahoma Health Department. The experiment oversampled ethnic and racial minorities such as African Americans, American Indians, and Hispanics. SEED OK design allows for establishing causality of any observed treatment/control differences in outcomes to the CDA policy intervention [32]. It also gives strong grounds to generalize results to the full Oklahoma state population. (See Figure 1 below for the randomization and intervention components of SEED OK.)

The SEED OK experiment uses a centralized account platform and the already existing financial structure of the 529 College Savings Plan, Oklahoma (OK 529), which helps with cost-efficiency and long-term sustainability [31]. The Oklahoma Treasurer’s office automatically opened a CDA for every child in the treatment group and deposited USD 1000 initial seed money. Families had the opt-out option. The USD 1000 deposits on behalf of the child beneficiaries in the treatment group were initially invested in the OK 529 Balanced Fund Option, which offered a mix of holdings in stock and bond funds. Funds in the state-owned accounts are now held in the OK 529 Moderate Age-Based Option [34]. The State of Oklahoma will send accumulated funds directly to the academic institutions when youth are involved in approved programs (e.g., in-state and out-of-state 4-year universities, community colleges, and vocational schools). Unused funds that remain in state-owned accounts after the beneficiary reaches age 30 will be returned to the state.

SEED OK offered two financial incentives to encourage the mothers of treatment children to open their own OK 529 accounts and make contributions from their own money in addition to opening the state-owned accounts. The financial incentive was a USD 100 initial deposit into an OK 529 account opened by a treatment family before a given date. Mothers who owned their own OK 529 accounts had the option to choose an investment that was more or less aggressive than the state-selected option and benefit from the state tax deduction.

The second incentive was saving matches for 4 years (2008–2011) to low- and moderate-income families in treatment groups that made deposits in mother-owned accounts. The experiment offered a 1-to-1 match rate for families with an annual adjusted gross income below USD 29,000 (up to USD 250 in matches per year) and a 0.5-to-1 match rate for those with an annual adjusted gross income between USD 29,001 and USD 43,499 (up to USD 125 in matches per year). To decide on the matching eligibility, Oklahoma State used public-assistance program records and the state tax-return data of participants. Account statements have been sent to treatment families in each calendar quarter between 2008 and 2019, and in each calendar year since. In the early years of the policy experiment, SEED OK also sent other program communication materials (e.g., letters and postcards), an additional mailing of two books to read with children, and a mailing of a T-shirt with the caption “Future College Graduate.” Members of the control group were eligible to open OK 529 accounts, but they did not receive the CDA intervention components. Evidence from SEED OK was instrumental in identifying ten key policy design elements, which are selectively used by countries with national CDA policies [32].

### 2.3. Asset-Effects Theory and SEED OK Research Findings

CDAs are based on the asset-effects theory that having assets, especially in the early years of life, positively affects child development and family well-being, parent–child interactions, and the education, health, and mental health of children and parents [26,35]. This theory posits that the impacts of assets extend far beyond their monetary value. The very existence of a designated account for a child’s future creates a psychological buffer, fostering a future orientation and a tangible sense of hope that reduce stress for parents [32]. This is because the account makes an abstract future goal, such as a college education, feel concrete and achievable. By providing a tangible stake in the future, the policy shifts parents’ mindsets, reduces the mental load of financial uncertainty, and encourages behaviors that align with these newfound, attainable aspirations.

These broader CDA impacts are explained by the convergence of several theoretical frameworks that articulate the causal mechanisms connecting CDAs to family engagement and child hope. At its core, asset-effects theory proposes the primary causal mechanism: the tangible asset itself creates a psychological shift in parents. By making the future feel more secure and achievable, the account directly fosters hope and a future-oriented mindset. This newfound optimism reduces present bias and motivates parents to engage in long-term planning and educational interaction with their children, seeing these activities as productive investments in an attainable goal.

Complementing this, the family stress model [36] explains the link between the CDA and parental capacity for engagement. By mitigating the chronic economic hardship and stress associated with saving for the future, the CDA frees up parents’ cognitive and emotional resources. This reduction in maternal depressive symptoms, as found in SEED OK, directly improves a parent’s ability to participate in positive, patient, and consistent educational engagement with their child. In turn, the family investment model [37] suggests that CDAs enable parents to increase their nonfinancial investments in their children. With a secure financial foundation for the future established, parents can shift their focus to investing time and emotional energy into their child’s development, such as helping with homework and discussing college plans. Finally, from a wider perspective, the dual-generation framework explains the synergistic and long-term effects. CDAs are an intervention that supports parents and children simultaneously. By empowering parents and reducing their stress (parent generation benefit), the policy creates a more stable and encouraging home environment that directly fosters children’s psychological well-being and hope (child generation benefit), disrupting intergenerational cycles of disadvantage. Together, these theories provide a comprehensive model where the CDA is the catalyst; it directly enhances parents’ capacity and motivation for educational engagement and boosts hope and reduces stress, creating a positive dual-generation feedback loop that supports child development.

The CDAs increased 529 account holding by 99 percentage points among children from low-income households (i.e., those with income below 200% of the federal poverty line), 98 percentage points among children of color, and 99 percentage points among children of mothers with less than a 4-year college degree.

Findings revealed that account holding and asset accumulation in the SEED OK CDAs impacted parental and child outcomes that were likely, in turn, to impact eventual enrollment and completion of postsecondary education, even before the actual distribution of accumulated funds. The data in the second wave suggest that the CDAs helped sustain the high educational expectations that treatment parents held for their newborn children [23], reduced the intensity of maternal depressive symptoms [38], and improved children’s early social-emotional development [39]. The sizes of CDAs’ effects on the outcomes of maternal depression, parenting, and children’s social-emotional development were similar to those of other early childhood interventions such as Early Head Start and Head Start [32]. The effect sizes for these three outcomes were also larger for SEED OK’s low-income subsample than for the full sample [32]. These findings suggest that the effects of a universal and automatic CDA policy on some social development outcomes are greater for disadvantaged families.

Building on prior SEED OK findings that CDAs positively impact early parenting and child development [31], this study extends the analysis into adolescence using data from Wave 3 of the experiment. We, therefore, hypothesize that the CDA intervention motivates parents to increase their educational engagement with their children over the long term. Consequently, we also hypothesize that children in the treatment group, benefiting from both the tangible asset and this sustained parental engagement, will report a more hopeful outlook toward their future compared to children in the control group.

## 3. Research Methods

### 3.1. Data and Sample

In 2007, the primary caregivers of 2704 children were selected at random from a probability sample of the 2007 newborns in OK to participate in the SEED OK experiment. The vast majority, over 99%, of these caregivers were mothers. Following the completion of a baseline survey conducted between fall 2007 and spring 2008, these caregivers (henceforth referred to as “SEED OK mothers” or simply “mothers”) were then randomly divided into a treatment group (n = 1358) and a control group (n = 1346) [40]. Approximately 84% (n = 2259) of these mothers successfully completed the Wave 2 survey during the spring of 2011, with 1149 in the treatment group and 1110 in the control group. Furthermore, comprehensive in-depth interviews of 60 participants conducted between mid-2009 and late 2010 provided valuable insights into the experiences and perspectives of SEED OK mothers regarding OK 529 [39].

Data collection for the Wave 3 survey was conducted between January and July 2020 [34], with approximately 67% of the mothers completing it by July 2020 (n = 1799; 921 in the treatment group and 878 in the control group). The outbreak of the COVID-19 pandemic brought about significant alterations in the daily routines, activities, and arrangements for all participants involved in the experiment. Notably, the closure of schools led to changes in parent–child interactions, particularly concerning educational activities. These changes, stemming from the pandemic, had a profound impact on schooling and home dynamics, ultimately affecting SEED OK children and their families, which in turn, may have influenced CDA outcomes. For instance, families coping with the stresses of pandemic life may have allocated less or no attention to their CDAs.

The onset of the pandemic necessitated several adjustments in the collection of survey data, including the closure of the survey call center, the suspension of in-person field visits, and the subsequent introduction and implementation of an online survey. These changes in the daily lives of SEED OK participants, alongside alterations in research methodologies, potentially influenced the impacts of CDAs and the accuracy of responses to surveys conducted after the pandemic began.

Consequently, we present findings from analyses utilizing both the full sample (n = 1425) and the pre-COVID-19 sample (n = 570). Data from the pre-COVID-19 sample were collected before the onset of the COVID-19 pandemic and the subsequent changes in research methodologies (this study utilizes 1 April 2020, as the demarcation date). The decision to present separate results is supported by three primary factors: firstly, the statewide closure of public schools in late March 2020 directly impacted the daily routines of families with children; secondly, the transition from telephone interviews to online surveys by the research team from this date; and thirdly, a sufficiently substantial sample size before COVID-19 facilitated statistical inference with adequate power.

### 3.2. Measures

#### Dependent Variable: Parent–Child Educational Engagement

In Wave 3 of our study, we assessed parent–child engagement in education by surveying respondents about the frequency of their communication with their children regarding school matters, academic progress, and future goals. Nine survey questions addressed these topics, such as how often adults inquire about upcoming assessments and how often children initiate conversations about postsecondary education. Details of these questions and their response options are provided in Table 1. The internal consistency of these items was considered acceptable (Cronbach’s Alpha = 0.76; [41]). To represent the overall level of engagement, we calculated a composite score by summing the responses to these nine questions (ranging from 8 to 35), with higher scores reflecting more frequent conversations.

### 3.3. Dependent Variable: Children’s Hope

A three-item scale developed by Child Trends assessed children’s hope. It asked respondents about children’s broad expectations of their future. This brief scale measured children’s expectation of positive experiences (expect good things to happen to them), excitement about the future (feel excited about what the future holds), and trust in a positive future (believe their future will be good). The scale demonstrates acceptable internal consistency (0.77) and produces a composite score ranging from 3 to 15, with higher scores indicating greater hope.

### 3.4. Independent Variables

The primary independent variable under investigation is participation in the SEED OK CDA intervention. Participants were assigned a code based on their group: one for the treatment group and zero for the control group. To account for potential influences on the outcome, we included various characteristics measured at the beginning of the study (Wave 1) for the child, mother, and household. Child: we controlled for age (in years), sex (male = 1, female = 0), and race/ethnicity (categorical variables representing different groups). Mother: we accounted for the mother’s age (in years), education level (categorical variables representing different levels), employment status (employed = 1, unemployed = 0), marital status (married = 1, other = 0), and self-reported health (ranging from 1 = poor to 5 = excellent). Household: we further controlled for homeownership (owned = 1, rented = 0), participation in government assistance programs (yes = 1, no = 0), primary language spoken at home (English = 1, other = 0), and geographic location (categorical variables representing different areas). Additionally, we included household size (number of residents) and income-to-needs ratio (calculated based on reported income and poverty guidelines). Further control variables included the mother’s reported parenting attitude (higher score = more positive) and saving habits (monthly saver = 1, nonsaver = 0).

### 3.5. Statistical Analyses

The primary goal of our analysis is to estimate the causal impact of the CDA intervention on parent–child educational engagement and children’s hope. Given that this study employs a rigorous, longitudinal randomized controlled trial (RCT) design—the gold standard for causal inference—our primary analytical strategy is multivariate linear regression [42]. This approach allows for a direct and powerful estimation of the average treatment effect. While other methods like propensity score matching are valuable for quasi-experimental designs, they are not necessary here [43], as the SEED OK experiment ensures comparability between groups through its robust randomization process, as demonstrated by the balance of baseline characteristics (see Table 2).

Our analyses employed multivariate linear regressions and path analysis [44,45] to evaluate the impacts of CDAs on both parent–child educational engagement and children’s hope. While randomization minimizes systematic baseline differences, we included a comprehensive set of control variables for child, mother, and household characteristics in our regression models [43]. This is a standard practice in RCT analysis to increase statistical power and control for any chance imbalances that may arise from sampling variation, thereby producing more precise estimates of the treatment effect. To ensure the stability and interpretability of our models, we conducted diagnostic tests for multicollinearity. Variance Inflation Factors (VIFs) for all predictors were well below the common threshold of 10, indicating that multicollinearity was not a concern in our models [46]. In the path analysis, we specifically examine whether the impacts of CDAs on children’s hope are mediated through parent–child educational engagement. All other standard assumptions for multiple linear regression (e.g., linearity, normality of residuals) were also checked and met.

Following the exclusion of observations with missing data, we conducted the aforementioned analyses on two distinct samples. Pre-COVID-19 Sample (n = 570): This analysis focused solely on the data collected before the significant impact of the COVID-19 pandemic on educational systems. Full Sample (n = 1425): This analysis included data from both pre- and post-pandemic periods, providing a broader picture of potential effects.

Given the existence of a pre-determined, theory-driven hypothesis regarding specific expected effects, we utilized one-sided *p*-values to assess statistical significance for the estimated impacts on both educational expectations and college preparation [47]. With this approach, we established a significance level of 0.10 for the one-tailed tests [48]. This approach is further supported by two key factors. Previous SEED OK research has consistently demonstrated positive educational impacts of CDAs on parents’ educational expectations, parenting practices, and college preparation [39]. This existing evidence strengthens our a priori hypothesis of positive effects, making one-sided tests appropriate. In addition, when utilizing the pre-COVID-19 sample (N = 570), the relatively small sample size reduces statistical power. Employing one-sided tests allows for a more sensitive detection of the hypothesized positive effects, given this limitation. All analyses were weighted to ensure their representativeness of the complete Oklahoma birth population across two specific 3-month periods within 2007: April–June and August–October [40].

We apply Confirmatory Factor Analysis (CFA) and Structural Equations Modeling (SEM) in supplemental analyses [49] to create alternative latent measures of our dependent variables and to examine the CDA impacts on parent–child educational engagement and children’s hope. The latent measures of parent–child educational engagement and children’s hope include all survey items listed in Table 1. The results of supplementary analyses are consistent with those generated from the main analyses proposed above and can be requested from the authors.

## 4. Research Results

### 4.1. Sample Description

Table 2 describes the characteristics of SEED OK children, mothers, and households by treatment and control status, for the pre-COVID-19 sample (n = 570). We found no significant differences between treatment and control groups, except for the treatment mothers’ higher likelihood of using English as their primary language and residing in metropolitan areas. Similar descriptive results (not reported) hold for the full sample (n = 1425). This suggests that, after 13 years, the randomization at Wave 1 still balances the treatment and control groups.

### 4.2. Bivariate Results: The Impact of CDAs on Parent–Child Engagement and Children’s Hope

Table 3 reports descriptive statistics of survey items for two dependent variables: parent–child educational engagement and children’s hope. In the pre-COVID-19 sample, the treatment group shows a consistent pattern of better outcomes across various survey items of two outcome measures compared to the control group. Specifically, in parent–child educational engagement, the treatment group demonstrates higher scores in study skills (2.82 vs. 2.76), grade checking online (2.78 vs. 2.70), homework completion (4.42 vs. 4.32), report card discussion (0.96 vs. 0.94), discussing jobs or careers with the child (3.02 vs. 2.89), and children’s initiated discussions on postsecondary education (2.66 vs. 2.59) and jobs (3.02 vs. 2.89). The treatment group has a total score of 25.80 with a standard deviation of 4.66, while the control group has a slightly lower total score of 25.30 with a standard deviation of 4.78. However, the treatment–control difference is only statistically significant on the measure of children’s-initiated discussion on jobs, as indicated by the bivariate one-tailed tests with a significance level of 0.10.

Regarding children’s hope, the treatment group in the pre-COVID-19 sample also exhibited higher levels of hope compared to the control group, with higher scores in expecting good things to happen (3.89 vs. 3.82), feeling excited about the future (4.14 vs. 4.00), and trusting the future will turn out well (4.22 vs. 4.11). The treatment–control differences in feeling excited about the future and trusting the future will turn out well are statistically significant (*p* < 0.10). The treatment group has a significantly higher total hope score of 12.24 with a standard deviation of 2.09, compared to the control group with a score of 11.94 and a standard deviation of 2.03.

In the full sample, although the two groups generally show similar results across most measures, the treatment group still maintains slightly better scores in some aspects, such as grade checking online (2.73 vs. 2.70), homework completion (4.31 vs. 4.27), discussing postsecondary education with the child (2.94 vs. 2.91), children’s initiated discussion on jobs (2.74 vs. 2.70), and feeling excited about the future (4.14 vs. 4.07), albeit without statistical significance. Overall, the findings suggest that CDAs positively influence parent–child educational engagement and children’s hope, with some improvements in pre-COVID-19 conditions across various dimensions of engagement and hope.

### 4.3. Multivariate Results: CDAs, Parental Educational Expectations, and Children’s Hope

Table 4 presents regression and path analysis results examining the impacts of CDAs on parent–child educational engagement and children’s hope for both the pre-COVID-19 sample and the full sample. The dependent variable is the total score of parent–child educational engagement for Model 1, and the total score of children’s hope for Model 2 and Model 3.

For the pre-COVID-19 sample, in Model 1, the treatment status variable shows a significant positive coefficient of 0.64 (*p* < 0.10), indicating that, compared to the control group, receiving the CDA intervention at birth is associated with higher levels of parent–child educational engagement in adolescence. This suggests that CDAs have a positive impact on enhancing parent–child educational engagement.

Transitioning to Models 2 and 3, which examine the relationship between CDAs and children’s hope, the coefficients of the treatment status remain significant. In Model 2, the coefficient for the treatment status is 0.28 (*p* < 0.10), indicating a positive impact of CDAs on children’s hope. In Model 3, which includes the treatment status variable alongside parent–child educational engagement, the coefficient for the treatment status reduces to 0.06 (*p* < 0.10), and the coefficient for parent–child educational engagement is significant, 0.30 (*p* < 0.001). These findings underscore the significant role of CDAs in shaping children’s hope, even when accounting for parent–child educational engagement. In the full sample, however, the treatment status variable is not statistically significant in all three models.

Overall, the results suggest that the treatment intervention positively influences parent–child educational engagement, which in turn may contribute to higher levels of children’s hopefulness.

## 5. Discussion

This study explores the psychosocial impacts of CDAs, using Wave 3 data from the SEED OK experiment. We investigate whether owning a tangible stake in their future fosters greater hope and encourages stronger parent–child educational engagement among adolescents. Analyzing data from the third wave of surveys in a longitudinal, randomized policy experiment with a statewide probability sample, the study reveals that accumulating assets through CDAs enhances parent–child educational engagement and fosters children’s hope for future development, as demonstrated by the pre-COVID-19 sample. Interpreted through the theoretical framework outlined earlier, these results offer experimental support for the mechanisms proposed by asset-effects theory [35]. Specifically, our findings suggest that by providing a tangible stake in the future, the CDA intervention reduces family stress and encourages parental investment in ways that positively alter both parental behaviors and children’s psychological outlooks, consistent with family stress and investment models [36]. Moreover, our findings extend the large body of correlational research on parental engagement [5,9] by providing causal evidence that a structural, asset-based intervention can be an effective lever for enhancing these crucial family processes. These findings also align with earlier SEED OK research on parental educational practices, parenting styles, children’s social-emotional development, and behavioral issues [39]. However, these novel findings provide empirical evidence of CDA effects on two new outcome measures: parent–child educational engagement and children’s hope during adolescence. Alongside impacts on other aspects of child development, the positive effects of CDAs on these new outcomes during adolescence support the asset-effect theory regarding nonfinancial impacts of asset accumulation for children, suggesting that such effects may persist from early childhood into adolescence.

In the multivariate analysis (Table 4), we observe that the sum score of parent–child educational engagement for treatment adolescents in the pre-COVID-19 sample was approximately 7% of a standard deviation (0.64) higher than that for counterparts in the control group. Similarly, the sum score of children’s hope for the treatment adolescents in the pre-COVID-19 sample was also about 7% of a standard deviation (0.28) higher than that for their counterparts in the control group. These findings indicate that parent–child educational engagement was more prevalent among the treatment adolescents, and they also held more positive expectations for their future. The regression coefficient of treatment status (0.07, *p* < 0.10) regarding the sum score of children’s hope was comparable to those of children’s gender (−0.09, *p* < 0.10) and household income-to-needs ratio (0.06, *p* < 0.11), emphasizing the noteworthy effect size of CDAs on these outcomes.

Moreover, the estimated size of the CDA’s effect on nonfinancial outcomes at ages 4 and 14, including parental educational expectations, parenting practices, children’s socioemotional development, and behavior problems, ranged from 8% to 16% of a standard deviation [39]. The positive effects on these nonfinancial outcomes found in both Wave 2 and Wave 3 suggest that the influences of this low-touch CDA policy on children and families were sustained, even though the funds in the treatment children’s CDAs had not yet been spent on postsecondary education.

Over the past decade, SEED OK has facilitated systematic research on the CDA policy model and its impacts on families and children [23]. This body of research has demonstrated that CDAs can have positive impacts on financial outcomes such as account holding and asset accumulation [32]. Additionally, SEED OK research has indicated that CDAs can help parents maintain high expectations for their children’s education [23], reduce the intensity of maternal depressive symptoms [39], decrease the use of punitive parenting practices, and improve children’s early social-emotional development [39].

The current study’s findings contribute to this growing body of research by demonstrating that CDAs can also have positive, long-term effects on parent–child educational engagement and children’s hope. These two effects should not be considered in isolation but rather as part of a larger set of findings on asset building’s long-term effects on children’s health, well-being, and development. The body of findings from SEED OK is consistent with the hypotheses of asset-effects theory [35]: that lifelong asset building can have long-term psychosocial and health benefits for families and children.

### 5.1. Implications for Policy and Research

These results have important implications for future research and policy aimed at promoting asset building for children’s health and well-being. Such understanding may contribute to the design and implementation of future CDAs in the United States and other countries. We note that the positive effects of CDAs on adolescent behavior problems stem from a specific form of universal and progressive CDA design, but effects may vary with differences in design. Findings on specific CDA effects on types of parent–child educational engagement could inform policy design improvements. If CDAs have stronger impacts on children’s future orientation and career preparation, some financial incentives in CDAs might be tied to specific children’s behaviors. Integrating career development services into asset-building policies could maximize CDA impacts on child development.

While the SEED OK experiment provides robust evidence within the U.S. context, it is crucial to consider that the policy’s effects may differ across cultural settings, limiting its external validity. The mechanisms through which CDAs influence family dynamics—such as fostering parental engagement and future hope—are deeply tied to cultural norms. For example, in more collectivist societies, the concept of individual asset-building may interact with family responsibilities and community expectations differently than in more individualistic cultures. The global expansion of CDA policies into countries such as Canada, Israel, Korea, Singapore, Uganda, and Kazakhstan [28] offers a valuable opportunity for future comparative research. Studying these implementations can help identify key cultural factors that mediate or moderate CDA impacts, paving the way for more culturally adaptable and effective policy models worldwide. Informed by the SEED OK policy design, seven states in the United States (California, Illinois, Maine, Nebraska, Nevada, Pennsylvania, and Rhode Island) have adopted statewide, automatic, universal CDA policies through legislation or administrative rule. These policies are very much in the development stage. The current study’s findings suggest that such statewide CDA policies may effectively promote positive parent–child interaction and enhance children’s future orientation for all children. Further research is needed to systematically examine the potential of universal CDA policy as a comprehensive system for adolescent development, including research on other aspects of child development not examined in the current study.

In summary, the findings carry broad implications across several domains. Theoretically, they provide additional evidence for asset-effects theory. Educationally, they demonstrate that a financial policy can directly influence family engagement processes crucial for learning. Socially, they suggest a structural approach to fostering hope and reducing psychosocial disparities. Finally, from a political standpoint, they underscore the potential of universal, asset-based policies to serve as an effective and potentially bipartisan platform for family and child support.

### 5.2. Limitations

Although SEED OK is exceptional as a randomized and long-running experiment with a statewide probability sample, the study’s findings should be considered in light of two key limitations. First, sample attrition, since the study’s inception reduced the participant pool by approximately 37%. While statistical reweighting was used to ensure the treatment and control groups remained comparable, this loss may limit the generalizability of our results.

Second, the Wave 3 data collection was disrupted by the COVID-19 pandemic, potentially affecting results for the full sample. These results indicated statistically nonsignificant impacts of CDAs on two examined outcomes. Therefore, our analyses focused on the results of the pre-COVID-19 sample. A similar strategy has been applied in other research [50]; however, the extent of the pandemic’s effects remains unclear. While our primary analysis focuses on the pre-COVID-19 sample for these reasons, it is important to consider the theoretical and policy implications of these nonsignificant findings. Beyond the methodological disruption, it is plausible that during a period of acute crisis, families’ attention shifted to immediate health and economic survival, potentially diminishing the perceived relevance of a long-term savings policy. Furthermore, because CDA funds are restricted for future use and cannot be accessed to mitigate immediate financial hardship, their psychological effect may have been muted for families experiencing acute pandemic-related stress. This context suggests that the nonsignificant effects in the full sample may reflect a temporary shift in family priorities during an unprecedented crisis, rather than a negation of the policy’s underlying impact in more stable conditions. Despite this, the consistently positive impacts of CDAs on nonfinancial outcomes, observed at Wave 2 and in the pre-COVID-19 sample, support the asset-effect hypotheses.

### 5.3. Conclusions

In summary, this study provides novel longitudinal evidence that CDAs positively impact parent–child educational engagement and adolescent hope. As millions of children worldwide gain access to these accounts [12,27], our findings underscore the policy’s role in not just building financial assets [34], but in shaping the psychological foundations for future success. This research supports a broader vision where asset-building for all children is a core strategy for fostering aspiration, strengthening families, and promoting equitable human development.

To leverage these findings and advance the CDA policy globally, several concrete actions can be taken. First, a global learning network for policymakers, researchers, and practitioners can be established to share best practices on CDA design, implementation, cultural adaptation, and scalable administrative platforms. Second, policymakers should formally integrate CDAs with existing social welfare policies and broader national strategies, such as financial inclusion and the UN Sustainable Development Goals, to position them as core tools for human capital building, poverty reduction, and equity. Finally, a standardized set of core evaluation metrics should be developed for cross-national comparison, capturing not only financial accumulation but also key psychosocial outcomes like hope and family engagement to build a robust global evidence base.

Overall, our study has yielded promising results, revealing the positive effects of CDAs on adolescents’ educational, social, and psychological well-being. These findings underscore the potential of CDAs as a policy tool not only for enhancing financial well-being but also for promoting positive human development among adolescents. In the larger picture, this evidence supports the broader significance of asset-building policies in nurturing resilient and healthy individuals, with very practical implications for enhancing the well-being of youth.

## Figures and Tables

**Table 1:** Survey Questions in Wave 3: Parent–Child Educational Engagement and Children’s Hope.

Survey Question	Response Categories
*Parent–Child Educational Engagement*	
In a typical month, how often would you say you or another adult in your household talked with the child about good study skills, like how to take notes, review for a test, or keep track of assignments?	1 (never)–4 (at least 3 times)
In a typical month, how often did you or another adult in your household check the child’s grade online?	1 (never)–4 (at least 3 times)
In a typical month, how often would you say you or another adult in your household asked the child if he/she had completed all homework?	1 (never)–5 (every school day)
In a typical month, how often would you say you or another adult in your household asked the child about upcoming tests or big assignments?	1 (never)–5 (every school day)
Last fall, when the child received his/her report card or final course grades, did you or another adult in your household discuss those grades with the child?	0 (No) and 1 (yes)
Last fall, how often did you talk with the child about his/her educational plans for after high school	1 (not at all)–4 (more than 4 times)
Last fall, how often did you talk with the child about careers or jobs he/she might be interested in	1 (not at all)–4 (more than 4 times)
Last fall, on his/her own, how often did the child talk to you about education or training after high school?	1 (never)–4 (More than 4 times)
Last fall, on his/her own, how often did the child talk to you about a job or career he/she would like to have when he/she grows up?	1 (never)–4 (More than 4 times)
*Children’s Hope Scale*	
The child expects good things to happen to him/her	1 (Not at all like)–5 (Exactly like)
The child feels excited about his/her future	1 (Not at all like)–5 (Exactly like)
The child trusts his/her future will turn out well	1 (Not at all like)–5 (Exactly like)

**Table 2:** Characteristics of the Pre-COVID-19 Sample (n = 570).

Characteristic	Control (*n* = 275)	Treatment (*n* = 295)
**Child**		
Male (%)	51.85	54.24
Race (%)		
Non-Hispanic White	67.67	71.46
Non-Hispanic African American	7.63	7.83
Non-Hispanic American Indian	7.65	9.00
Non-Hispanic Asian American and Pacific Islander	2.45	2.43
Hispanic	14.60	9.28
Child’s age, M (SD), by year	14.49 (0.19)	14.47 (0.18)
**Mother**		
Age, *M* (SD), by year	26.74 (5.66)	26.77 (5.50)
Education (%)		
Below high school	13.75	12.52
High school	31.67	26.61
Some college	24.04	28.29
4-year college or above	30.54	32.58
Health status, M (SD)	4.07 (0.89)	4.22 (0.88)
Marital status (% married)	68.93	69.52
Employment status (% working)	57.40	58.96
**Household**		
Household size, M (SD)	2.91 (1.12)	3.17 (1.31)
Save monthly (% yes)	68.78	64.36
Parenting attitude index, *M* (SD)	10.98 (1.38)	11.04 (1.38)
Homeownership (% yes)	55.64	55.33
TANF participation (% yes)	6.13	8.14
SNAP participation (% yes)	28.27	27.88
Income-to-needs ratio, M (SD)	265.10 (277.35)	255.89 (248.97)
English as primary language at home (% yes) *	90.42	95.53
Geographic areas (%) *		
Metropolitan area	63.68	72.57
Micropolitan area	19.46	17.74
Other	16.86	9.69

Note. Results are weighted. Two-tailed statistical tests. TANF = Temporary Assistance for Needy Families; SNAP = Supplemental Nutrition Assistance Program. * *p* < 0.10.

**Table 3:** Parent–Child Educational Engagement and Children’s Hope.

	Pre-COVID-19 Sample		Full Sample	
Survey Items	Control (*n* = 275)	Treatment (*n* = 295)	Control (*n* = 709)	Treatment (*n* = 716)
*Parent–Child Educational Engagement*				
Study skills	2.76 (0.85)	2.82 (0.85)	2.81 (0.82)	2.81 (0.82)
Grade checking online	2.70 (1.00)	2.78 (0.95)	2.70 (0.95)	2.73 (0.93)
Homework	4.32 (1.05)	4.42 (0.97)	4.27 (1.07)	4.31 (1.01)
Upcoming tests	3.51 (1.16)	3.46 (1.12)	3.58 (1.09)	3.53 (1.10)
Report card	0.94 (0.24)	0.96 (0.20)	0.95 (0.22)	0.95 (0.23)
Talk with the child about educational plans for after high school	2.69 (0.93)	2.79 (0.91)	2.70 (0.93)	2.74 (0.90)
Talk with the child about careers or jobs	2.90 (0.92)	2.90 (0.90)	2.85 (0.93)	2.84 (0.91)
The child talks about education or training after high school	2.59 (1.06)	2.66 (1.00)	2.62 (1.03)	2.61 (1.00)
The child talks about a job or career	2.89 (0.98)	3.02 * (1.00)	2.91 (0.98)	2.94 (1.00)
Total parent–child educational engagement score	25.30 (4.78)	25.80 (4.66)	25.39 (4.75)	25.46 (4.71)
*Children’s Hope*				
Expect good things to happen	3.82 (0.93)	3.89 (0.89)	3.89 (0.94)	3.82 (0.94)
Feel excited about the future	4.00 (0.84)	4.14 * (0.85)	4.07 (0.88)	4.06 (0.89)
Trust the future will turn out well	4.11 (0.76)	4.22 * (0.78)	4.17 (0.79)	4.15 (0.84)
Total children’s hope score	11.94 (2.03)	12.24 * (2.09)	12.14 (2.16)	12.03 (2.21)

Notes. Results are weighted. We conduct bivariate one-tailed tests with a significance level of 0.10 to compare differences between the treatment and control groups. * *p* < 0.10.

**Table 4:** Multiple Regression and Path Analysis Results for Parent–Child Educational Engagement and Children’s Hope.

Variable	Pre-COVID-19 Sample (*n* = 570)			Full Sample (*n* = 1425)		
	Model 1	Model 2	Model 3	Model 1	Model 2	Model 3
Treatment Status	0.64 * (0.45)	0.28 * (0.17)	0.60 * (0.04)	0.10 (0.28)	−0.12 (0.11)	−0.03 (0.03)
Parent–child educational engagement			0.30 *** (0.04)			0.26 *** (0.03)

Notes. Results are weighted. We conduct one-tailed tests with a significance level of 0.10. The dependent variable of Model 1 is the total score of parent–child educational engagement. The dependent variable of Model 2 and Model 3 is children’s hope. Results on control variables are not reported in the table. * *p* < 0.10, *** *p* < 0.01.

## Data Availability

The datasets presented in this article are not readily available because the data are part of an ongoing study. Requests to access the datasets should be directed to Dr. Jin Huang (jhuang23@wustl.edu).
